# Circadian Clock Genes in Colorectal Cancer: From Molecular Mechanisms to Chronotherapeutic Applications

**DOI:** 10.3390/biomedicines14010110

**Published:** 2026-01-06

**Authors:** Haoran Wang, Jieru Zhou, Suya Pang, Yiqing Mei, Gangping Li, Yu Jin, Rong Lin

**Affiliations:** 1 Division of Gastroenterology, Union Hospital, Tongji Medical College, Huazhong University of Science and Technology, Wuhan 430022, China; u202113242@hust.edu.cn (H.W.); zjr17798263006@163.com (J.Z.); psy265047@163.com (S.P.); 17302297637@163.com (Y.M.); selinalin35@hotmail.com (R.L.); 2 Tongji Medical College, Huazhong University of Science and Technology, Wuhan 430030, China

**Keywords:** circadian clock, chronotherapy, circadian genes, colorectal cancer

## Abstract

Colorectal cancer (CRC) is a life-threatening malignancy, but our understanding of its pathogenic mechanisms remains incomplete—posing a major constraint on the development of effective therapeutic strategies. The transcription-translation feedback loop of clock genes (e.g., *BMAL1*, *CLOCK*, *PER1/2/3*, and *CRY1*/*2*) provides a promising novel avenue for deciphering the initiation and progression of CRC. Mounting evidence indicates that core circadian clock genes play pivotal roles in CRC oncogenesis by orchestrating the regulation of the cell cycle, epithelial–mesenchymal transition (EMT), metabolic reprogramming, and the tumor microenvironment. This review systematically summarizes the expression patterns and mechanistic roles of core clock genes in CRC, while elucidating their molecular underpinnings in tumor progression via key signaling cascades (e.g., Wnt/β-catenin and c-Myc/p21 pathways). We emphasize the associations between circadian disruption and CRC—including diagnostic markers, prognostic assessment, and chemosensitivity—and provide an in-depth discussion of chronotherapeutic strategies and their translational potential. Finally, we identify unaddressed scientific questions and propose future research directions to facilitate the development of novel targeted therapies for CRC.

## 1. Introduction

Colorectal cancer (CRC) is the third most common cancer worldwide, and the severity of this disease continues to increase with age [1]. However, the mechanisms of pathogenesis and development remain to be fully elucidated.

**Figure 1:**
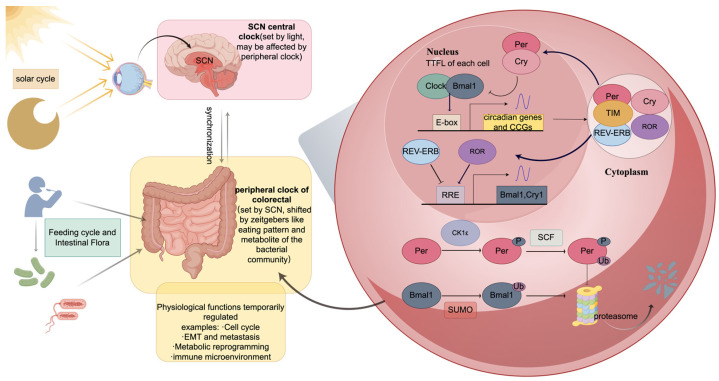
Environmental cues regulate the transcription-translation feedback loop of the circadian clock via direct and indirect mechanisms. Light directly stimulates the Suprachiasmatic Nucleus (SCN), altering the central circadian clock. In contrast, food modulates intestinal flora and gastrointestinal cells, whose secretions thereby regulate the colorectal peripheral clock and influencing the synchronization between the central and peripheral clocks.

Recently, associations between clock genes and the initiation and progression of CRC have attracted increasing interest. The circadian rhythm is indispensable to living organisms. It is created endogenously by genetically encoded molecular clocks to regulate relevant downstream programs throughout the body and optimize many biological processes through a transcription/translation feedback loop (TTFL) [2,3,4], like in Figure 1. *Basic helix–loop–helix ARNT like 1* (*BMAL1*) and its partner, *circadian locomotor output cycle protein kaput* (*CLOCK*), are the main regulators of the loop; they work as a transcription factor that drive the positive arm of the loop by heterodimerization. Binding to the E-box element in gene promoters, this transcription factor regulates numerous clock-controlled genes (CCGs), including their suppressors, the *Period* (*PER*) and *Cryptochrome* (*CRY*) family members, which constitute the negative arm of the loop [2,3,4]. Moreover, *REV-ERB* (also known as *nuclear receptor subfamily 1 group D member 2*, *NR1D2*), *retinoic acid receptor-related orphan receptor α* (*RORA*), *casein kinase 1ε* (*CK1ε*), and *TIMELESS* (*TIM*) have also been shown to modulate the loop at various levels [5].

In summary, as core positive regulators of the TTFL pathway, *BMAL1*/*CLOCK* can regulate downstream *PER*/*CRY*/*TIM* feedback components, thereby modulating other downstream genes and related physiological processes. Thus, disorders of circadian rhythm may affect a wide range of ailments, e.g., caners, especially CRC [6,7,8]. Numerous hallmarks of CRC, including cell cycle dysregulation, tumor metastasis, metabolic reprogramming, and the tumor microenvironment are closely linked to clock genes [5,9,10,11].

Emerging evidence has demonstrated that dysregulation of clock genes exhibits cancer-type-specific patterns [12]. In this review, the mechanisms by which clock genes participate in the tumorigenesis, development, and progression of colorectal cancer are systematically reviewed and summarized, focusing on key pathological hallmarks as well as their clinical implications for molecular diagnosis and the development of novel therapeutic strategies.

## 2. Role of Clock Genes in CRC Pathology

Irregular artificial light stimulation would cause light disruption, resulting in abnormal sleep patterns (e.g., variable sleep onset latency and duration), and it is widely recognized as the primary mechanism affecting the circadian clock [13,14]. Furthermore, recent studies have supported the idea that irregular eating patterns (e.g., irregular time and amount of food) and even DNA damage also participate in the development of CRC [6,15,16]. Notably, diet-induced disruption of peripheral clock in colorectal tissue may potentially affect the circadian rhythm stability of other peripheral organs (e.g., liver) via pathways such as cancer cell metastasis, and even feedback to disrupt central clock at the genetic level [17]. Both eating and sleeping patterns contribute to CRC development by disrupting clock gene expression [15,18,19], as illustrated in Figure 1, leading to various pathological manifestations [7,12].

### 2.1. Disruption of the Cell Cycle in CRC

Cell cycle disruption is a hallmark of cancer cells. Both the cell cycle and the biological clock exhibit successive phases of transcription/translation, protein modification, and degradation [20]. Recent findings have indicated an increasingly significant role for clock genes in the regulation of the cell cycle [12,21,22]. Specifically, in CRC, clock gene dysregulation disrupts the Wnt/β-catenin pathway, leading to cell cycle dysregulation [23,24].

The Wnt pathway serves as a key intercellular coupling component linking intestinal circadian rhythms to the cell cycle. However, the tumor suppressor effect of *PER2* on the Wnt pathway varies across different animal models. In in vitro cell models and mouse models, *PER2* has been identified as a tumor suppressor gene, as it maintains the normal expression rhythms of *c-Myc* and *Cyclin D* [24]; in contrast, *PER2* deficiency in zebrafish xenograft models and *PER2* knockout in *Drosophila* do not induce carcinogenesis [25,26]. This discrepancy may be attributed to two factors: first, the presence of other *PER* family isoforms in zebrafish and *Drosophila* which compensate for the cyclin-related regulatory function of human *PER2* (e.g., *BmPER*); second, the relatively complex *PER2*-related signaling pathways in mammals. This also suggests that for studies exploring the role of clock genes in human diseases, mammalian models are preferred to ensure the consistency of gene function. Notably, *PER2* exhibits relatively consistent functions in maintaining the stability of the cell proliferation cycle.

*TIM* functions as an oncogene, and is highly expressed during the S and G2 phases of DNA replication and cell division. Its depletion induces G2-phase cell cycle arrest in human colorectal cancer cell lines (e.g., HCT116). Notably, widespread *TIM* overexpression is observed in clinical colorectal cancer specimens. These observations are closely associated with *TIM*’s involvement in regulating the phosphorylation of key cell cycle kinases, including CDK1. Additionally, *TIM* may also function to maintain genomic stability [27]. Moreover, the combination of Wee1-like protein kinase or CHK1 inhibition resulted in a cumulative decrease in CRC metabolism, with little effect on normal human colonic epithelial cells [27], which indicates that *TIM* is promising therapeutic target for the treatment of CRC. Recently, poly ADP-ribose polymerase (PARP) inhibitor forms a *TIM*, PARP polymer to prevent replisome transcription-replication conflicts (TRCs) [28]. This confirms that *TIM* acts as one of the therapeutic targets.

*BMAL1* and *CLOCK* exert negative regulatory influences on the cell cycle. The heterodimer can bind to the E-box region of the *c-Myc* promoter and directly repress its transcription to further upregulate *CHK2*, *p53*, and Wee1 [24]. Upregulation of Wee1 activates Cdc2, leading to phosphorylation of the CDK1/cyclin B complex and subsequent stagnation of cancer cells at the G2/M stage [24], which may result in poorer outcome. Additionally, *BMAL1* maintains the normal self-renewal balance of intestinal stem cells (ISCs) by inhibiting excessive activation of the Hippo signaling pathway, e.g., regulating Yap nuclear localization activity. Its depletion induces abnormal activation of the Hippo pathway, accompanied by downregulation of the Wnt pathway, and subsequently triggers cell cycle dysregulation and abnormal excessive proliferation of ISCs [29]. Although other studies have reported contradictory effects of *BMAL1* on ISC proliferation [30], this discrepancy may stem from differences in study model conditions, such as non-cancer context, and ISC subpopulation focus. Nevertheless, all these studies highlight the indispensable core role of *BMAL1* in maintaining ISC proliferation homeostasis.

*CRY* may function as a tumor promoter in CRC. Overexpression of *CRY1* and *CRY2* leads to an increased proportion of S-phase in CRC cells and inhibits cell apoptosis [31]. The underlying mechanism could be affecting APC/C interactions and mitotic checkpoint complex formation [32]. Of interest, these phenomena have not been validated in intestinal models.

Overall, the regulatory role of clock genes in the cell cycle of CRC has been partially elucidated. Through multiple signaling pathways such as Wnt and key molecules including p53 and Wee1, they collectively maintain cell cycle homeostasis. Dysregulated expression of clock genes thus leads to cell cycle dysregulation, which in turn promotes CRC tumorigenesis and progression. Currently, further exploration of the underlying mechanisms in this field still faces several challenges. First, although the core function of clock genes in stabilizing the cell cycle is generally conserved across non-mammalian models, there may be significant differences in the details of regulatory mechanisms. Therefore, if non-mammalian models yield conclusions inconsistent with those from mammalian models, research on this pathological mechanism should primarily take the results of mammalian models as the core reference. Second, research on some members of the clock gene family remains largely incomplete. Existing evidence indicates strong correlations between the regulatory mechanisms of different clock genes. Filling these research gaps will therefore provide important support for the development of this field.

### 2.2. Tumor Metastasis of CRC

Another important feature of cancer is the ability of cells to migrate and undergo several steps to achieve metastasis. Previously, distant metastases were present in 15–25% of CRC patients at diagnosis [33], and new distant metastases occurred in 18–25% of patients within five years of diagnosis [34]. Recently the anticancer rate for metastatic CRC still decreased, and relative survival has not improved significantly [35]. The 5-year survival rate for those who develop liver and lung metastases without resection is 2.6%, and they are more likely to experience serious adverse events [36]. These findings suggest that metastatic CRC is a serious problem that urgently needs to be addressed.

#### 2.2.1. Metastasis Associated with EMT

Recent studies have concluded that epithelial-mesenchymal transition (EMT) is inextricably linked to cancer invasiveness and metastasis and that EMT is involved in the induction of drug resistance in CRC, which is a topic of interest in this field. By targeting the EMT-associated gene, *metastasis-associated in colon cancer 1* (*MACC1*), clock genes can regulate the EMT process; in particular, one study found that knockdown of *BMAL1* and *PER2* significantly increased *MACC1* levels and CRC invasiveness [37].

*PER2* was found to repress the expression of these EMT genes by recruiting the multimeric nuclear proteins EZH2 and SUZ12 as well as HDAC2 to the octameric transcription factor 1 binding site of the *TWIST1* and *SLUG* promoters [38].

*TIM* inhibits CRC metastasis. Multiple clinical studies confirm that *TIM* expression level is negatively correlated with CRC metastatic potential. In the zebrafish xenotransplantation model, *TIM* knockdown significantly enhances the in vivo metastatic potential of CRC cells. The nude mouse model further uncovered the molecular mechanism underlying this phenomenon: *TIM* depletion reduces the expression levels of epithelial markers (e.g., E-cadherin/CDH1) significantly, while upregulating the expression of mesenchymal markers (e.g., Vimentin, FN1) and the EMT core transcription factor ZEB1. This thereby drives the EMT process and ultimately enhances the invasive ability of CRC cells [39]. Although research on ZEB1-targeted immunotherapeutic strategies for CRC has been reported, ZEB1 expression is regulated by multiple upstream factors (e.g., transcription factors like SNAIL), and this may increase the complexity of targeted intervention. Thus, developing targeted strategies for *TIM*, the upstream regulator of ZEB1, may be a more promising direction. Compared with ZEB1, *TIM* has a simpler expression regulatory network, is less disturbed by other irrelevant factors, and can more precisely affect the immune microenvironment and malignant phenotype of CRC by regulating the *TIM*-ZEB1 axis [40].

#### 2.2.2. Metastasis Associated with Angiogenesis

Another molecule that has been confirmed to be associated with CRC metastasis is vascular endothelial growth factor (VEGF). Clock genes work as its modulators, involved in the metastasis mechanism. *BMAL1* could stimulate tumor angiogenesis and metastasis, even chemotherapy resistance via binding the E-box of *VEGF* [9,41,42]. And *CLOCK* stimulates these pathological processes via interacting with HIF-1α/ARNT to activate the expression of VEGF, thereby indicating the potential for a novel antimetastatic therapeutic approach.

Elevated levels of *CRY* expression have been demonstrated to be associated with increased rates of proliferation and migration, along with decreased overall survival rates in patients with CRC [20]; however, the mechanism remains unclear.

In summary, the evidence suggests the multifaceted involvement of clock genes in the processes of invasion and metastasis in CRC. One study even confirmed that when *BMAL1* is highly expressed, the heterodimer formed with *CLOCK* binds to the E-box elements in the promoter region of the *Rab27a* gene derived from human CRC cells, thereby transcriptionally activating the expression of *Rab27a*; this process promotes the secretion of exosomes by CRC cells, and these exosomes can further enhance the migration ability of CRC cells and vascular endothelial cells (e.g., HUVECs) in the tumor microenvironment, creating favorable conditions for the invasive metastasis of CRC. For therapy, the efficacy of employing chitosan and HA to modify nanoparticles, thereby enhancing cargo transport and reducing the risk of CRC, has been tested, identified as a potential means to inhibit EMT and reverse CRC resistance [43]. Of interest, given the established relationship between metastasis and chemotherapeutic resistance, the study of clock genes related to metastasis could offer significant advancements.

### 2.3. Metabolic Reprogramming of CRC

The metabolic profiles of cancer cells, such as those associated with glycolysis, lipid metabolism, glutaminolysis and oxidative phosphorylation, as well as changes in mitochondrial kinetics, are known to be altered. This altered profile is a consequence of the reprogramming of cellular metabolism to meet the need for dysregulated metabolism and proliferation [44,45]. A well-known example of a change in cellular metabolism in tumors is the predominant use of glycolysis even in the presence of oxygen, referred to as the Warburg effect [46]. Gene expression, the tumor microenvironment, and the treatment response can be profoundly affected by such changes, leading to tumor metastasis and adverse results. Defining the reprogramming mechanism and targeting altered molecules will aid in the development of clinical therapies [44,45]. Recently, some studies have shown that the clock genes are also involved in the development of CRC by regulating the metabolism of tumor cells [12].

#### 2.3.1. Glucose Metabolism

Levels of multiple glycolysis-related genes and metabolites exhibit circadian fluctuations [10,47]. This rhythmic characteristic has been confirmed to be regulated by clock genes [48].

Specifically, *CK1δ*/*ε* enhances the stability and activity of p53 by phosphorylating multiple sites (e.g., Ser-6, Ser-9, Ser-15, and Ser-20). It then directly inhibits aerobic glycolysis by upregulating the downstream target gene *TIGAR*, reduces glucose uptake by suppressing *GLUT1* expression, and decreases the shunting of glycolytic intermediates to the pentose phosphate pathway by inhibiting G6PD activity, ultimately comprehensively suppressing the aerobic glycolysis process in CRC cells [49]. As a specific inhibitor of *CK1δ*/*ε*, IC261 is expected to serve as an effective therapeutic agent in relevant clinical scenarios for CRC [49].

Moreover, the core clock gene *BMAL1* plays a negative regulatory role in the glycolytic metabolism of CRC. As mentioned earlier, *BMAL1* deletion mediates the activation of the Wnt signaling pathway, which further upregulates downstream c-Myc to drive enhanced glycolysis—this effect has been validated in mouse intestinal organoids and CRC patient-derived organoids. The TCGA-COAD database further confirms that CRC patients with this molecular signature (aberrant activation of the Wnt-c-Myc-glycolysis axis) have significantly shorter overall survival (Log-rank *p* = 0.0032) [50], suggesting that clock gene-mediated regulation of the Wnt pathway may be a core pathological pathway in the initiation and progression of CRC.

Hexokinase HKDC1 is implicated in various gastrointestinal tumors and participates in tumorigenesis by regulating glucose metabolism [51]. *BMAL1* and HKDC1 exert mutual inhibition: perturbed *BMAL1* expression induces time-dependent changes in HKDC1 levels and metabolism, characterized by increased glycolytic activity, enhanced cellular energy supply, and a shift toward a metastatic phenotype. In metastatic CRC cells, the inhibitory effect of *BMAL1* on HKDC1 is markedly attenuated, and the expression of clock genes and their regulatory pathways differ somewhat between primary and metastatic CRC cells [10]. Thus, in mechanistic studies on clock genes and CRC metabolic reprogramming, it is crucial to focus on the heterogeneity of gene expression and regulatory mechanisms induced by metastasis. Notably, HKDC1 is also involved in processes such as immune evasion [52]; therefore, *BMAL1*-mediated modulation of the HKDC1-related glycolytic pathway may be essential for CRC patients whose metabolic and metastatic properties are associated with immune evasion.

#### 2.3.2. Lipid Metabolism

Dysregulated cholesterol metabolism is noteworthy in CRC, and *RORα*/*γ* play pivotal roles as cholesterol–nuclear receptors to activate the transcription of the ubiquitinating enzyme NEDD4 and subsequently promote c-Myc degradation, leading to the therapeutic strategy of combining RORα/γ agonists and atorvastatin in CRC to inhibit metastasis and proliferation [53].

#### 2.3.3. Other Metabolic Pathways

In addition, other metabolic pathways are potentially linked to the circadian clock. For example, *CRY1* knockdown induces 8-h rhythms in amino acid, methylation, and vitamin metabolites, decoupling metabolites from transcriptional rhythms and potentially affecting nutrient sensing in vivo [54]; however, these rhythms have not been directly demonstrated in CRC models. Although there are still many gaps in the mechanism of metabolic reprogram of CRC, especially in lipid metabolism, these findings provide the possibility of further drug development and better therapeutic strategies in conjunction with therapies.

### 2.4. Tumor Microenvironment of CRC

Another profile of tumors is immune evasion [12], and disturbances in the balance between immune promotion and immune suppression lead to the escape of cancer cells from immune control, resulting in tumorigenesis and tumor progression [55]. This process is usually mediated by the tumor microenvironment, which consists of the extracellular matrix (ECM) and a variety of cells, particularly immune-associated cells [56]. Circadian regulate the tumor microenvironment by regulating immune cells, etc., and their dysregulation leads to adverse outcomes, such as tumor metastasis, and determines the efficacy of immunotherapy [57,58,59,60]. These evidence reveal the feasibility of immune-conjugated chronotherapy. The exploration of the correlation between the tumor microenvironment and clock genes is needed to establish a foundational framework for the enhancement of therapeutic strategies and the mitigation of adverse prognoses.

#### 2.4.1. Alterations in the Tumor Microenvironment Unrelated to Enterobacteria and Their Metabolites

As previously stated, the disruption of circadian genes has been demonstrated to directly promote CRC. In some analyses of the genomic and transcriptomic landscapes of clock genes, alterations in clock genes are significantly correlated with those of immune-related genes, thereby regulating immune cells within the context of CRC [11]. This, in turn, has implications for CRC metastasis. Moreover, these alterations hold promise as markers for specific immune subtypes within the context of CRC [61].

Focusing on the specific mechanism, *BMAL1* knockout has been demonstrated to increase the expression of c-Myc and downstream inflammatory cytokines, such as Cxcl5, through the upregulation of Wnt signaling [62], simultaneously reducing IL-33 and leading to the reduction in PDL1-expressing Breg cells (regardless with or without rhythmic oscillations), resulting in a lower level of CD4+ T cells, ultimately promoting CRC [63]. In addition, these effects elicit increased neutrophil mobility and myeloid-derived suppressor cells (MDSCs) aggregation. These *BMAL1*-related mechanisms result in enhanced inflammation and consequent immunosuppression in the TME, though PD-L1 expression exhibits conflicting trends across different studies [61,63,64]. Notably, both opposing trends converge on the conclusion that *BMAL1* exerts an anti-inflammatory role.

In the DSS-induced mouse colitis model, *REV-ERBα* inhibits the NLRP3 inflammasome via a dual mechanism: first, it specifically binds directly to the inflammasome’s promoter region to suppress transcription; second, it inhibits p65 and its downstream NF-κB pathway, indirectly repressing NLRP3 and attenuating macrophage-mediated inflammatory responses. The anti-inflammatory effect of the *REV-ERBα* agonist SR9009 in this pathway has been validated in animal models [65]. Based on this mechanism, a nanolipid carrier (NLC) hydrogel enriched with high galacturonic acid pectin (i.e., modified citrus pectin, MCP4) and loaded with 6-gingerol (6G) has been developed. It targets inflammatory sites and downregulates NLRP3 activation by regulating the NF-κB inflammatory pathway and *REV-ERBα*/*β* [66].

Suppressed expression of inflammatory factors, adhesion molecules, and NF-κB was found in *CRY1*/*CRY2*-overexpressing endothelial cells, whereas the opposite result was obtained in deletion cells [67]. *CRY1* binds to adenylate cyclase to limit cAMP production, and its absence leads to increased cAMP levels resulting in sustained activation of protein kinase A (PKA) signaling, which leads to increased inflammation [67]. Different types of immune cells are co-regulated by their intrinsic circadian clocks and environmental stimuli, and exert their functions by participating in various signaling pathways; these pathways mediate inflammatory responses through direct or indirect effects on immune cells or related cytokines.

Of interest, many studies merely associate changes in the number or activity of immune cells with alterations in the expression of clock genes, without further exploring the underlying mechanisms. Harnessing omics to unravel the hidden information in these studies would be of great benefit to clarify drugs targeting clock genes and key downstream molecules such as NLRP3, subsequently optimize chronotherapy.

#### 2.4.2. Tumor Microenvironment Associated with Enterobacteria and Their Metabolites

Although significant progress has been made in research on the regulatory mechanisms of non-intestinal-specific immune cells, studies on the CRC microenvironment need to incorporate a key factor unique to the intestinal system: the role of the gut microbiota [68]. Dysregulation of the gut microbiota and its metabolites (e.g., taurocholic acid (TCA)) interacts with the circadian rhythm to trigger inflammatory responses, and microenvironmental changes may be an important link in the pathogenic process of CRC, thereby affecting CRC progression, treatment response, tumor metastasis, and clinical prognosis [69,70].

Notably, gut microbiota dysbiosis can independently disrupt local intestinal clocks, and many of its derived metabolites inherently exhibit distinct circadian rhythms are significantly dysregulated in CRC. Under physiological conditions, the levels of short-chain fatty acids (SCFAs) oscillate circadianly, peaking during the dark phase in mice, and it is synchronized with the rhythmic expression of intestinal clock genes (e.g., *BMAL1*, *PER2*); as a secondary bile acid metabolized by the microbiota, TCA’s diurnal fluctuations are also tightly coupled to the intestinal circadian rhythm [69]. In contrast, feeding during the rest period (a factor that directly induces microbiota dysbiosis) can alter the phase of the colonic peripheral circadian clock and cause microbiota dysbiosis in mice, reducing SCFA-producing bacteria and butyrate levels, which in turn decreases Treg cell density, leads to increased intestinal barrier permeability, and promotes CRC development [6]. This indicates that changes in microbiota composition can independently affect the clock rhythm of intestinal epithelial cells through metabolite mediation, without relying on central clock regulation. Meanwhile, CRC-related microbiota dysbiosis (e.g., reduced abundance of Bifidobacterium and Lactobacillus) can disrupt the intrinsic circadian oscillations of intestinal immune cells (e.g., macrophages); for example, decreased butyrate impairs the rhythmic expression of *BMAL1* and *REV-ERBα* in colonic macrophages, triggering sustained inflammation, which echoes the core mechanism of *REV-ERBα* regulating macrophage inflammation [65]. These changes not only decouple microbial metabolites from the host circadian rhythm but also exacerbate pathological processes by regulating immune cell function: For instance, TCA can epigenetically promote glycolysis in MDSCs, enhance the monomethylation of the target gene *H3K4*, and inhibit CHIP-mediated ubiquitylation of PDL1, leading to the aggregation of MDSCs and dysfunctional CD8+ T cells in the lungs of mice. This weakens tumor-specific immune function and promotes CRC progression and lung metastasis [70,71].

Overall, gut microbial oscillations may be an important factor associated with the host circadian rhythm and tumor microenvironment, and there are both interacting links and independent regulatory components between the gut microbiota and circadian rhythms. However, microbiome science is still in its early stages of development, and its application in CRC faces numerous challenges: the lack of longitudinal population cohorts with stool samples and sufficient clinical metadata to support relevant analyses, the need for in-depth exploration of the causal relationships between the microbiota, its metabolites, and various pathological processes, as well as the significant impact of experimental factors such as inter-individual heterogeneity in microbiota composition and sampling time on research reproducibility [68]. These are all long-term issues that need to be addressed in this field in the future.

## 3. Clinical Application of Clock Genes in CRC

The role of clock genes in CRC has been extensively studied, and they form a complex molecular timing system that coordinately controls multiple cellular processes associated with CRC development. Moreover, disruption of clock genes is closely associated with poor chemotherapy response and adverse prognosis [72]. Even tumor stage, subtype and survival time can be predicted with clock genes [73]. Based in these findings, some advanced applications and treatment strategies have been developed.

### 3.1. Clock Genes in CRC Diagnosis and Prognostic Tests

**Figure 2:**
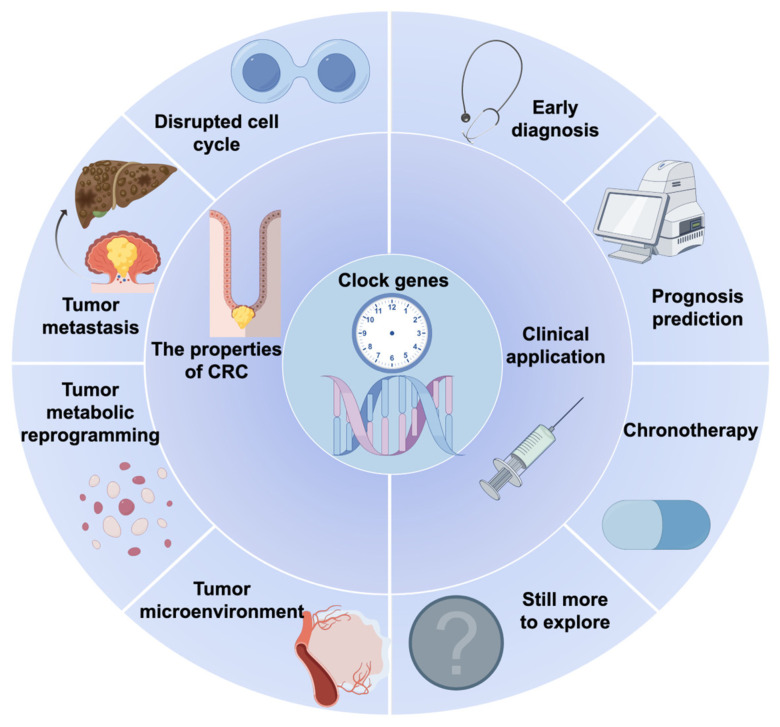
Summary and Perspectives of the Circadian Clock in the Pathogenesis and Clinical Treatment of CRC.

Multiple analyses based on TCGA database have confirmed that various clock genes are closely associated with the prognosis of cancer patients like in Figure 2.

Although there are currently no specialized prognostic prediction analyses for clock genes in CRC, multiple studies on non-small cell lung cancer and other cancers have revealed certain commonalities; core clock genes such as *BMAL1* and *PER2* show a positive correlation between their expression levels and patient prognosis, while the expression level of the *TIM* gene exhibits a negative correlation with prognosis. As diagnostic and prognostic prediction indicators, clock genes exhibit good accuracy, with the area under the curve (AUC) reaching 0.8 in some analytical models, which is superior to traditional detection indicators (e.g., CEA, CA199, and CA125) [74,75]. The expression of clock genes in CRC exhibits location-related heterogeneity. Existing study has shown that the expression difference of *CRY1* has more significant prognostic predictive value in female patients. Specifically, in female patients with right-sided colon CRC, the expression level of *CRY1* in tumor tissues is significantly higher than that in adjacent tissues, and this high *CRY1* expression is negatively correlated with patient prognosis; in contrast, no significant expression difference of such clock genes (i.e., *CRY1*, *CRY2*) is observed between tumor tissues and adjacent tissues in female patients with left-sided CRC [76]. In summary, by detecting clock gene expression levels, we may obtain multiple types of information, such as the TNM stage of CRC, degree of malignancy and the prognosis to a certain extent. Although there is still a problem of an insufficient sample size for testing, further expansion of the number of clinical tests will better extract useful information from the clock gene level and provide more accurate and simple diagnostic methods.

Recent finding has also revealed that specific circular RNAs (*hsa_circ_0049487*, etc.) are associated with the adenoma-carcinoma transition in CRC, and CRC exosome-derived *hsa_circ_0003270* also has increased sensitivity in diagnosing CRC and is highly correlated with lymph node metastasis [77]. This finding reveals that circRNAs may serve as potential biomarkers for early diagnosis. Considering that clock genes regulate the release of exosomes and downstream RNA expression [41], using genomics, transcriptomics, and methods such as correlation analysis to discover the axis of clock genes to mRNAs or exosomes involved in the development of CRC will not only help to explore the mechanism of clock gene regulation in CRC pathology but also further improve CRC-specific detection indices. Quantifying the association between the disordered expression of clock genes and the occurrence of CRC, the use of a multi-gene panel to detect the abnormal expression of clock genes may be a powerful tool for future CRC detection.

### 3.2. Clock Genes in CRC Chronotherapy

In clinical practice, it has been identified that the pharmacologic effects of anticancer drugs significantly vary with respect to the time of administration [78,79]. In accordance with the role of clock genes in pathological processes and overall prognostic performance, such as the correlation of high *BMAL1* expression levels with increased oxaliplatin sensitivity, suggesting the potential application of clock genes in cancer, new therapies combining clock genes are emerging [73]. All these factors lead to chronotherapy, a therapeutic approach that adjusts the timing of chemotherapeutic drug administration according to biological rhythms, primarily the circadian clock, as illustrated in Figure 2. The proportion of cells in the DNA synthesis phase exhibits a 24 h circadian rhythm, with peak levels observed between 8:00 and 20:00 in various tissues such as bone marrow and intestines. Meanwhile, the activity of the rate-limiting enzyme for fluorouracil metabolism, dihydropyrimidine dehydrogenase (DPD), peaks between 22:00 and 00:00. This circadian characteristic enables daytime administration to maximize the inhibitory effect on cancer cell proliferation [80]. Therefore, aligning drug administration with the 24 h rhythms of host drug metabolism-related enzyme activities and target cell killing mechanisms not only enhances therapeutic efficacy but also reduces tumor drug resistance while minimizing toxic side effects on normal tissues.

The therapeutic superiority of chronotherapy has been demonstrated in the clinic. For example, 5-FU or capecitabine with continuous chronotherapy has been demonstrated to enhance drug tolerance and efficacy while concomitantly reducing mucosal toxicity in cancer patients [81]. Moreover, chronotherapy with oxaliplatin, fluorouracil, and folinic acid has been utilized in the treatment of metastatic CRC [80]. The results revealed that the objective response rate in the chronotherapy group was 51%, which was significantly greater than the 29% reported in the constant-rate infusion group. In addition, the objective response rate significantly reduced the incidence of severe mucosal toxicity and peripheral neuropathy [79], which allows for surgery in patients with unresectable liver metastases after receiving chronotherapy and leads to 39–50% 5-year survival [82], resulting in great improvement of the efficacy of CRC treatment. Moreover, some studies have also focused on the combination of the circadian clock and ICB therapies. For mCRC, conventional immunotherapy has brought the longest currently known survival extension for patients [83,84]. However, the regimen combining chronotherapy with immunotherapy such as administering anti-PD-L1 drugs based on the abundance rhythm of MDSCs has demonstrated superior therapeutic efficacy [62]. In recent years, the majority of research has focused on the construction of a series of chronotherapy models, with studies examining the role of various parameters in treatment, including sex and the Cyclin/Cdk complex, among others, in improving therapeutic outcomes [85,86,87].

Currently, there are relatively few reports on drug administration experiments conducted in accordance with the theory of chronotherapy, and there is a lack of suitable rhythmic biomaterials and systems. Furthermore, while there is some commonality in the role of the parameters suggested by the existing different models, the differences also reflect the lack of a standardized model for the evaluation and testing of chronotherapy. For example, individuals with different endogenous circadian clocks (e.g., morning types and evening types) should be grouped for discussion as much as possible, and each group should be given the most appropriate exogenous regulatory factors (e.g., food, light) instead of applying uniform timing for all. Meanwhile, attention should be paid to using minimally invasive alternative samples during sample collection to ensure patient compliance when collecting samples at different time points. Moreover, differences in the timing of radiotherapy and chemotherapy make it difficult to establish uniform criteria for evaluating efficacy. The general lack of consideration of sex variables and lack of information on samples obtained at different time points in randomized clinical trials makes the accuracy of the results questionable, which need to be fully considered. Finally, computer algorithms should be optimized to further reduce biases caused by samples. In addition, the effectiveness of the use of the biological clock to control cancer and the effects of cancer-related treatments on the biological clock remain unclarified [20]. In summary, although the superiority of chronotherapy for clinical treatment has been verified in some models, the optimization of its application still needs to be further explored.

Despite evidence has emerged demonstrating that adaptive immune responses are less robust during evening hours than during daytime hours following initial stimulation, offering novel insights into the clinical utilization of drugs and their applications [88,89], the underlying mechanism remains incompletely clarified. However, in some existing models, the underlying mechanism by which the expression levels and rhythms of clock genes and drug treatment timing synergistically enhance therapeutic efficacy has been partially elucidated. In 5-FU chemotherapy, *BMAL1* levels peak during the dark phase of mouse, and the activity of 5-FU metabolic enzymes regulated by *BMAL1* (such as UMPS, UCK2, and UPP2) also exhibits a highly synchronized circadian rhythm. Administering 5-FU during this time period significantly enhances its therapeutic efficacy [72]. Similarly, the expression rhythm of *PER1* shows high synchronization with that of DPD during the dark phase [90]. Moreover, the *BMAL1*/*CLOCK* transcriptional complex has been demonstrated to reduce the sensitivity of B cells to CY treatment by increasing the survival of cyclophosphamide (CY)-metabolite-responsive B cells. *CRYs*, in turn, function as a negative regulator of *BMAL1*, thereby reducing the survival of CY-metabolite-responsive B cells, subsequently improving the efficacy of CY treatment [91]. *PER1* was recently shown to induce high levels of glycolysis, leading to trastuzumab resistance by increasing HK2 expression upon interaction with PPARγ [92].

Notably, there are significant differences in the rhythmic expression of genes among the four consensus molecular subtypes (CMS1–4): the peak of circadian oscillation in CMS1 is concentrated at the 9 h phase with higher *TIM* gene expression; in contrast, the peak of circadian oscillation in CMS4 is enriched at the 3 h phase, characterized by low *TIM* expression and high ZEB1 expression [93]. This difference not only explains the high metastatic potential of CMS4 observed clinically but also indicates that chronotherapeutic strategies should vary among different subtypes.

## 4. Conclusions and Outlook

Clock genes’ role in CRC has been partially clarified. Disrupted expression rhythms of clock genes directly trigger a series of pathological alterations as illustrated in Table 1, making them potential biomarkers for CRC therapeutic intervention [6,74,75,94]. Additionally, combining classic CRC treatments (e.g., chemotherapy, immunotherapy) with chronotherapy significantly improves efficacy and reduces toxic side effects, with promising preliminary evidence as illustrated in Table 2 [62,79,80,81,82,85].

Notably, two under-explored but translationally valuable connections deserve attention: first, the synergistic effect of gut microbiota-based interventions and chronotherapy facilitates clinical translation, as time-restricted feeding (TRF) reshapes colonic circadian rhythms by regulating microbiota composition (e.g., restoring SCFA-producing bacteria) [6], while supplementing SCFA-producing probiotics/prebiotics (e.g., Bifidobacterium breve, inulin) restores microbial metabolite and intestinal clock rhythms; second, epigenetic modifications (e.g., methylation, histone acetylation) targeting clock genes regulate downstream processes (e.g., metabolism, cell cycle), representing a promising research direction [102,103].

Future research should focus on two core directions: first, identify key functional nodes in the pathological process by which clock genes regulate CRC initiation and progression, and develop synthetic biology-based technologies to achieve direct targeted therapy against clock gene-related targets; second, clarify the circadian clock’s impact on traditional drug therapy mechanisms, and optimize administration protocols and treatment processes via rhythm regulation to enhance the precision and effectiveness of CRC treatment.

Clock genes have demonstrated great potential in CRC diagnosis, treatment, and regimen optimization. However, three core bottlenecks must be addressed. At the animal models level, prioritize mammalian models and correct circadian active period differences between animals and humans to ensure clinical applicability; at the detection and clinical management level, refine efficacy indicators to a precise temporal dimension and strengthen patient adherence to rhythmic regimens to avoid efficacy impairment from temporal deviations; at the research design level, incorporate circadian heterogeneity-related factors (e.g., gender, age, CMS) into clinical cohort analyses to clarify their regulatory effects on treatment outcomes, laying a foundation for personalized plans.

Breaking through these bottlenecks will bring a revolutionary leap to CRC diagnosis and treatment.

## Figures and Tables

**Table 1:** Summary of the Roles of Clock Genes in CRC.

Clock Genes	Downstream Effect	Expression Level in CRC	Target	Reference
*BMAL1*	Cell cycle arrest	N/A	Wee1	[58]
		Decreased	yes-associated protein 1	[29]
	Promotes or inhibits tumor metastasis	N/A N/A	MACC1 exosome	[37] [95]
	Inhibits metabolic reprogramming (reduces aerobic glycolysis)	Decreased	HKDC1, other metabolism-associated genes	[10]
		Decreased	c-Myc	[54]
	Maintains a noninflammatory tumor microenvironment and immune cells healthy	Decreased	IL-33, PDL1	[63]
	Associated with the sensitivity of chemotherapeutic agent (5-FU, and CY)	N/A	5-FU chronotherapy related genes, such as UPP2, UCK2 and UMPS	[72]
		N/A	CY-metabolizing B cells	[91]
*CLOCK*	Cell cycle arrest	N/A	Wee1	[58]
	Promotes tumor metastasis	Upregulated	HIF-1α, ARNT, VEGF	[9]
*PER1*/*2*/*3*	Cell cycle arrest	Decreased	c-Myc, cyclin D1	[24]
		Upregulated	c-Myc, Wee1	[25]
		Decreased	CLK/CYC	[35]
	Inhibits tumor metastasis	N/A	MACC1	[37]
		Decreased	TWSIT1, SLUG, SNAIL1	[38]
*CRY1*/*2*	Promotes cell cycle	N/A	Wee1	[58]
	Promotes tumor metastasis	Upregulated	unclear	[20]
	Associated with the location of CRC	N/A	unclear	[76]
		N/A	CY-metabolizing B cells	[91]
*TIMELESS*	Promotes the cell cycle	Decreased	CHK1, CDK1, Wee1	[27]
	An appropriate concentration would promote tumor	Decreased (facilitates EMT)	ZEB1	[39]
		Upregulated (facilitates EMT)	Myosin-9	[96]
*CK1ε*	Inhibits metabolic reprogramming (reduces aerobic glycolysis)	Upregulated	P53	[51]
*REV-ERBs*	Promote or inhibit tumor metastasis	Decreased	SNAIL1	[25]
		N/A	MACC1	[37]
	Maintain a noninflammatory tumor microenvironment and immune cells healthy	Decreased	P65, Nlrp3	[66]

**Table 2:** Chronotherapy in Multiple Therapy.

Therapeutic Regimens	Clinical Research Methods	Outcomes	Limitation	Cancer Type	Reference
Cyclophosphamide	Nonclinical research	*BMAL1*/*CLOCK* transcriptional complex reduce the sensitivity of CY treatment by elevating the survival of cyclophosphamide CY-metabolite-responsive B cells	Animal model results may not be directly applicable to humans. Incomplete coverage of tumor types	N/A	[91]
Trastuzumab	Nonclinical research	*PER1* induces high glycolysis and expression level of HK2, subsequently leading to trastuzumab resistance	Results may not be directly applicable to humans	Gastric cancer	[92]
ICIs	Nonclinical research	A higher rate of survival was observed in subjects who received ICIs in the morning compared with those who received it in the afternoon.	Animal model results may not be directly applicable to humans. Incomplete coverage of tumor types	Various cancer types	[58]
Irinotecan, Oxaliplatin, fluorouracil	Prospective study (Phase II)	Chronotherapy demonstrated improved efficacy with reduced hematologic toxicity	Small sample size. Lack of a control arm	mCRC	[97]
Oxaliplatin, Fluorouracil, Folinic Acid	Prospective study (Phase III)	Objective response was 51% versus 29% in the chronotherapy group and constant-rate infusion group, respectively, and lower neuropathy and mucosal toxicity was observed in the chronotherapy group	Small trial size. Different rate of surgery of metastases in two arms	mCRC	[81]
Nivolumab Pembrolizumab Atezolizumab	Retrospective study	Infusions at least 20% of ICI after 16:30 had a statistically significant shorter median PFS but no difference in OS	Small sample size variation in ICIs	NSCLC	[94]
Ipilimumab, Nivolumab, Pembrolizumab	Longitudinal study	Adaptive immune responses are not as strong in the evening to an initial stimulus as they are in the daytime	Lack of a control arm	Melanoma	[88]
Melatonin	Prospective study	The combination of chemotherapy and melatonin resulted in superior one-year survival and tumor regression rates.	The association with the circadian clock remains to be elucidated. The patients might have been melatonin-deficient	Various cancer types	[98]
LYC-55716 (cintirorgon)	Prospective study (Phase I)	The RORγ agonist works as an effective therapeutic reagent	Excessive variation exists among patient demographics.	Various cancer types	[99]
Angiotensin-converting enzyme inhibitors, angiotensin-II receptor blockers	Prospective study	The administration of antihypertensive medications prior to bedtime results in substantial improvements in blood pressure control and a reduction in adverse outcomes compared with the administration of these medications after waking.	The population is limited, and the applicability of the conclusions to younger groups is uncertain.	Hypertension	[100]
Temozolomide	Prospective study	A greater duration of survival was observed in subjects who received treatment during the morning hours as opposed to the evening hours.	Lack of a control arm. A comparison between the chronotherapy group and the general treatment group was not performed	Glioblastoma	[101]

mCRC, metastatic colorectal cancer; ICI, immune checkpoint inhibitor; NSCLC, non-small cell lung cancer; PFS, progression-free survival.

## Data Availability

No new data were created in this review.

## Abbreviations

The following abbreviations are used in this manuscript:

CRC	Colorectal cancer
EMT	Epithelial-mesenchymal transition
TTFL	Transcription/translation feedback loop
CCGs	Clock-controlled genes
*BMAL1*	*Basic helix–loop–helix ARNT like 1*
*CLOCK*	*Cycle protein kaput*
*PER*	*Period*
*CRY*	*Cryptochrome*
*NR1D2*	*Nuclear receptor subfamily 1 group D member 2*
*RORA*	*Retinoic acid receptor-related orphan receptor α*
*CK**1ε*	*Casein kinase 1ε*
*TIM*	*TIMELESS*
SCN	Suprachiasmatic Nucleus
EKs	Extracellular regulated protein kinases
CHK1	Checkpoint kinase 1
CDK1	Cell cycle protein-dependent kinase 1
PARP	Poly ADP-ribose polymerase poly ADP-ribose polymerase
TRCs	Transcription-replication conflicts
ISCs	Intestinal stem cells
MACC1	Metastasis-associated in colon cancer 1
CBP	CREB-binding protein
VEGF	Vascular endothelial growth factor
ECM	Extracellular matrix
MDSCs	Myeloid-derived suppressor cells
NLC	Nanolipid carrier
6G	6-gingerol
PKA	Protein kinase A
SCFAs	Short-chain fatty acid
AUC	Area under the curve
TCA	Taurocholic acid
DPD	Dihydropyrimidine dehydrogenase
CY	Cyclophosphamide
CMS	Consensus molecular subtypes
mCRC	Metastatic colorectal cancer
ICI	Immune checkpoint inhibitor
NSCLC	Non-small cell lung cancer
PFS	Progression-free surviva
TRF	Time-restricted feeding
